# Stapled Peptides—A Useful Improvement for Peptide-Based Drugs

**DOI:** 10.3390/molecules24203654

**Published:** 2019-10-10

**Authors:** Mattia Moiola, Misal G. Memeo, Paolo Quadrelli

**Affiliations:** Department of Chemistry, University of Pavia, Viale Taramelli 12, 27100 Pavia, Italy; mattia.moiola01@universitadipavia.it (M.M.); misalgmemeo@gmail.com (M.G.M.)

**Keywords:** stapled peptide, structurally constrained peptide, cellular uptake, helicity, peptide drugs

## Abstract

Peptide-based drugs, despite being relegated as niche pharmaceuticals for years, are now capturing more and more attention from the scientific community. The main problem for these kinds of pharmacological compounds was the low degree of cellular uptake, which relegates the application of peptide-drugs to extracellular targets. In recent years, many new techniques have been developed in order to bypass the intrinsic problem of this kind of pharmaceuticals. One of these features is the use of *stapled peptides*. *Stapled peptides* consist of peptide chains that bring an external brace that force the peptide structure into an α-helical one. The cross-link is obtained by the linkage of the side chains of opportune-modified amino acids posed at the right distance inside the peptide chain. In this account, we report the main stapling methodologies currently employed or under development and the synthetic pathways involved in the amino acid modifications. Moreover, we report the results of two comparative studies upon different kinds of stapled-peptides, evaluating the properties given from each typology of staple to the target peptide and discussing the best choices for the use of this feature in peptide-drug synthesis.

## 1. Proteins

Proteins, the building blocks of life, play a pivotal role in all the aspects of the cellular systems, from structural stabilization to the transport of nutrients, ions and small molecules, from immune defence to enzymatic activities. These macromolecules are displaced in all cells, in every part of them and, as suggested by their name, from the Greek word πρωτειoς that means “*of main importance*”, they are essential for the life of every living organism. Not only the big proteins such as enzymes are fundamental molecules for living organism but the small peptides, constituted by less than 50 amino acids, also play some pivotal roles, such as hormones, antibacterial agents, neurotransmitters, and so forth [1].

The first classification of these molecules dates back to the XVIII century, by the studies of Antoine Fourcroy [2] and, few years later, by the analysis of Gerardus Johannes Mulder [3] and Jons Jacob Berzelius [4], that assigned the name “Proteins” to this class of macromolecules.

However, the complexity of these molecules, their variety and the difficulty in studying the cellular environment did not allow an easy recognition and analysis of these macromolecules. Only with the development of more accurate analytical and biological techniques permitted in the 1949 the first resolution of protein structure with the description of the Haemoglobin and Myoglobin structure by Max Perutz e Sir John Cowdery Kendrew [5]. From that moment, the development of the analytical and synthetic techniques for proteins and the introduction of computational methods capable to predict the possible structures of a molecule, permit the extensive study of these essential biological compounds.

During the years, the medicinal chemists did not just watch at the proteins as target compounds for possible pharmacological treatments but tried to use this class of compounds as drugs for the treatment of a great variety of disease—from metabolic diseases to haematological pathologies and even to cancer treatment [6]. In fact, short peptide chains play crucial roles in the organisms, acting especially as messengers for most endocrine signals, such as for the G protein-coupled receptors or the growing factor ones. This key role has been exploited in order to obtain drugs that act as agonists in the signal transduction processes [7].

The use of peptides in the pharmacological field has some advantages—they display a fast systemic absorption that can avoid the first-pass metabolism; the targeting can be precisely controlled and the pharmacokinetic can be easily tuned by structural modifications [8].

However, they turn out to be quite unstable in the cellular environment and they can be easily hydrolysed in acidic or enzymatic conditions. Moreover, their molecular weight is quite high and so the access to the cellular environment is more difficult. In addition, the numerous synthetic steps required for the synthesis of peptides make hard to obtain large quantities of these compounds [8].

Despite these limits, the development of this class of drugs in recent years is growing strongly—it is estimated that the peptide-based drugs market is more than 40 billions of dollar per year, about the 10% of the global drugs market and it is growing faster than the one of any other kind of drugs [6].

### Peptides as Drugs

In the first half of the 20th century the study of natural peptides, in order to comprehend their biological role and their structure, played a pivotal role in the development of technologies that later became fundamental for the research of new drugs. A clear example of how peptides played a very important role as pharmacological principle at the beginning of the 20th century was the isolation [9] and the subsequent commercialization of Insulin, in 1923, when the structure and pharmacology of this peptide-hormone were not yet known [10].

However, in the following years, the research of peptides as possible pharmacologically active compounds aroused less and less interest, because it has been preferred to focus the general attention on the research of biologically active “small molecules” instead of peptide-based drugs [6].

This was determined by several factors—a greater simplicity of synthesis made the small molecules economically more competitive, while at the same time, the disadvantages of peptide-based drugs seemed to largely outweigh their advantages.

**Figure 1:**
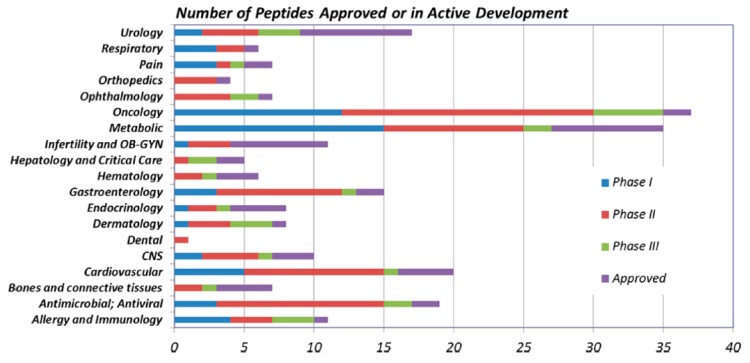
Peptides approved and in active development by therapeutic area (2016) [6].

Despite this, in the last two decades the research of peptide-based drugs has resumed strength, driven by the innovations in the field of molecular biology and genetic engineering that have allowed an easier synthesis of polypeptides and, at the same time, by the discovery and analysis of new natural peptides that have found applications as drugs. In the last 20 years, 28 new peptide-based drugs have been approved worldwide and many others are still in development—more than 200 peptides are still in the preclinical stage, while about 170 are involved in various stages of clinical tests in order to be approved as drugs especially in the field of metabolic disease and oncology (Figure 1, Table 1) [6].

Research on new peptide-drugs employs two opposite strategies—the first, in chronological order, consists of the manipulation of a known, well-studied natural peptide, in order to enhance or tune its pharmacological and pharmacokinetic properties, maintain its natural target of action; the second, less common, consists of the synthesis and then the screening of large libraries of peptides, evaluating their pharmacological properties, in research of a new biologically active peptide [11].

Peptide-based drugs are often easier to functionalize than other pharmaceuticals, offering good chances to tune their properties and target selectively the desired objective. They display low levels of immunogenicity, along with non-significant hepatic metabolism and low level of drug-drug toxicology [8,12]. All these properties can now be exploited due to an increasing automation of the synthetic pathways of peptides, which allows to small group of researchers the development of peptide-based drugs, while small molecules usually require bigger research team.

Although in the last years the development of new peptide-based drugs has become more and more important, peptide pharmaceuticals still have some disadvantages in respect of the small molecule drugs.

Peptides are in fact membrane impermeable—they are not able to pass the cellular membrane and so their target are limited to the extracellular or transmembrane space. Moreover, peptides are not able to pass even the intestinal mucosa and so it is not possible to employ the oral route of administration for this kind of drugs. This is due to the fact that the bioavailability of orally-administered pharmaceuticals is strictly linked to the molecular weight of the bioactive compound—if the compound have a molecular weight higher than 500–700 Daltons, its bioavailability, if orally-administered, is minimal and decreases as the mass increases. For this reason, the most common oral route of administration had to be replaced by alternative routes, such as subcutaneous or intravenous ones [8].

Besides the intestinal membrane, the peptides cannot also cross the blood-brain barrier. This fact limits the use of peptides as neuro-drugs but, at the same time, it allows the use of this class of compound without the fear of neurotoxic side effects [8].

Peptide drugs are also biologically unstable, precisely because of their biological role. Lots of peptides play as hormones or other regulatory molecules that bind selectively their target receptors when needed and that at the same time can be quickly removed when their request is expired. For this purpose, a very precise regulation system is involved in balancing and feedback loops that control the synthesis, release and degradation of these compounds. It determines a short plasma half-life for most of peptides, which are cleared primarily by proteolytic degradation and then removed by renal filtration, leading to some pharmacokinetic problems [8].

As mentioned earlier, the synthesis is a crucial step for the development of peptide pharmaceuticals and it probably represents the main problem for these drugs. The classical chemical synthesis, in fact, relies on a very long series of steps involving protection, deprotection and activation of amino acidic functional groups. This is due to the fact that every amino acid contains both a nucleophilic functional group, the amino one and an electrophilic one, the carboxylate and also a side chain, the properties of which depend on the nature of the amino acid [13].

The principal method of synthesis consists of the solid-phase supported synthesis, which involves the use of a porous solid support, usually an -NH${}_{2}$ or -OH functionalized resin, on which the first amino acid of the chain is linked by the condensation of the carboxylic moiety. The solid matrix that supports the synthesis plays a pivotal role in the synthesis—in fact, after each reaction, a purification step is needed, in order to remove the non-reacted molecules and the by-products [13]. The solid support allows a very easy workup, which consists of the simple washing of the matrix with a solvent that removes all the compound that are not linked to the resin, enhancing the yield of reaction and limiting the waste of solvents, time and other things required for more complex types of purification.

The chemical synthesis of peptides also permits obtaining a peptide containing unnatural or modified amino acids, but it is limited to polypeptide chains of a maximum of ca. 50 amino acids because of the high number of synthetic steps that limited the yield of the desired product.

For the synthesis of bigger polypeptide chains, such as proteins, recombinant methods are used [14]. These methods consist of the use of genetic-modified organism in which a part of DNA from another source has been inserted in his genetic material (so called recombinant DNA). The recombinant method allows to obtain complex proteins without the difficulties of a long chemical synthesis but it is limited to natural peptides because is not possible to insert unnatural amino acids or peptidomimetic bonds. One clear example of recombinant protein is the recombinant Insulin, that from 1982 replaced the original animal-derived compound—the human Insulin gene was inserted in *E. Coli* or *Saccharomyces cerevisiae* DNA, in order to produce Insulin for human use [10].

Nevertheless, in the next future will be probably possible to use recombinant synthesis on large scale, with the possibility to also insert non-natural or modified amino acids [15].

In order to enhance the properties of stability and resistance to hydrolysis of peptide chains, it is possible to insert some changes in the structural conformation of the amino acids or in the polypeptide itself [6]. First of all, it is necessary to determine which amino acidic residues are strictly fundamental for the desired pharmacological effect—this can be done by the positional scanning of the peptide, consisting of the substitution of each side chain with the smallest alternative that retains a similar conformation and then evaluating its importance in the biological activity.

For the proteolysis protection, it is possible to functionalise the extremes of the peptide chain, if these are not involved in binding interactions; the easier modifications consist of the acetylation of the N-terminus and the transformation of the C-terminus in a primary amide.

The use of D-amino acids can also prevent the peptide degradation, especially by chymotrypsin, conferring protection to the nearby residues.

**Figure 2:**
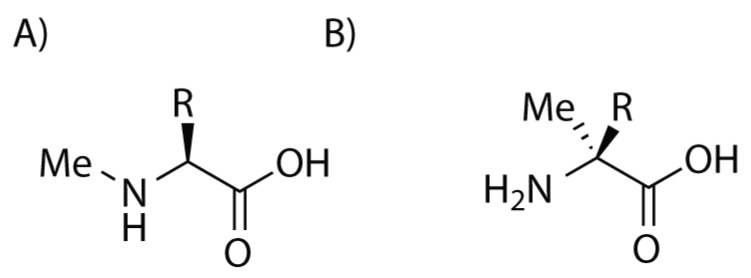
Example of methylation of the N-terminus (**A**) and of the α-carbon (**B**).

Another possible strategy in order to enhance the resistance of the peptide is to use α,α-disubstituted-amino acids, especially α-methyl-amino acids (Figure 2). These compounds protect the nearby amino acidic residues in a similar manner to D-amino acids but at the same time they do not display the risk of disrupting interference with the other side chains. In fact they preserve their original spatial disposition and they result to be confined in even more strictly conformations that appear to be useful in some context, especially for helical peptides. Unfortunately, the hindrance of the methyl group on the stereogenic centre requires harsher conditions for the coupling of these amino acids. The methylation of the amino group of each amino acid can protect the peptide from hydrolysis, improve its solubility and, moreover, in some cases it can lead to the formation of membrane-permeable peptides, that could be administered orally (Figure 2). However, the introduction of a methyl group on the nitrogen atom disrupt its hydrogen bond interactions, with a loss of activity.

The side chains of the peptide, especially if not involved in binding, can also be modified in order to tune the peptide properties, especially the proteolytic stability and the solubility. The latter can be enhanced by the replacement of the unnecessary hydrophobic regions and the modulation of its isoelectric point, in order to have the right-charged amino acid at the desired pH.

**Scheme 1:**
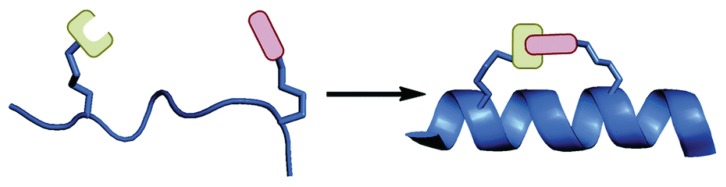
Example of peptide stapling [16].

The most promising technique to stabilize peptides seems to be the cyclization of linear peptides. This method is employed especially for helical peptides and takes the name of *peptide stapling* (Scheme 1). It consists of the linkage between two side chains of the same peptide that maintain and stabilize the helical structure of the chain. The external brace limits the flexibility of the molecule, reducing the entropic penalty of binding and orientation of the peptide and it improves affinity and selectivity to the target. Moreover, this conformation hides the amide bond inside the helix, enhancing the protease stability and allowing an easier permeation of the cellular membrane [16].

## 2. Stapled Peptides

**Figure 3:**
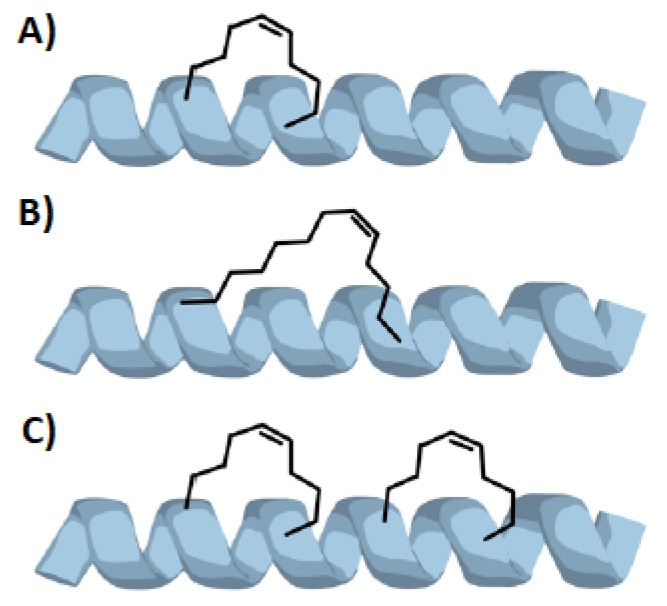
Example of *i, i + 4* (**A**), *i, i + 7* (**B**) and double *i, i + 4* (**C**) stapling [17].

As previously mentioned, in order to force a peptide to assume a α-helical structure it is possible to link together the side chains of two amino acids forming a stapled peptide. The number of stapling bridges inside the same peptide is not limited to one—for long peptides it is possible to use a double stapling, using four amino acids in order to get two different side braces (Figure 3C) [16]. However in literature triple (or more) stapled peptides are not listed, probably because a double stapling gives generally enough helicity to the desired peptide that is not necessary a further stapling.

As expected, is necessary to choose accurately the position of the stapling brace.

First of all, it is fundamental that the two (or more) amino acids involved in the stapling are at the right distance one from the other, with side chains that can form a brace of the right lenght. Usually, the residues are located in position *i* and *i + 4* (*i + 3*) or *i + 7*. For the *i, i + 4* stapling, 7–8 atom chains are generally used in order to form a brace between the two stereogenic carbon atoms; while for *i, i + 7* stapling 10–12 atom chains are employed, in many cases with the aid of a D-amino acid at one side of the brace [16,17,18,19].

It is also necessary that the side brace does not interfere with the activity of the peptide, disrupting fundamental interactions with its target substrate. Moreover is better to not involve charged amino acid side chains in the stapling in order to not change the overhaul net charge and charge distribution of the peptide. Walensky et al. recommend to put the stapling chain between the hydrophilic and hydrophobic faces of the future α-helix, in order to not disturb its amphipathic character [20].

Another possibility is to use the stapling bridge in order to stabilize the weak points of the helix, such as helical turn that contains proline or glycine residues. It is also important to remember that peptide stapling is efficient only with peptides that are substantially α-helices in their natural context. In fact, without this natural folding propensity is probable that the side chains necessary to the formation of the brace may never juxtapose sufficiently to react [17].

### 2.1. The α-Helix

The α-helical structure is one of the most common secondary structure in proteins and peptides. It is constituted by a right-handed spiral, maintained by hydrogen bond interactions between the amidic hydrogen of an amino acid in position *i* and the carbonyl group of the amino acid in the position *i + 4* of the peptide chain. Usually about ten amino acids are involved in the formation of the helix but they can range from a minimum of four to even forty amino acidic residues [1].

This kind of structure is not the only helical structure that can be found in proteins—also a left-handed helix (especially for D-amino acid peptides or for glycine-rich peptides) or other kinds of spirals such as the 3${}_{10}$- (H-bond between position *i* and *i + 3*) and the π-helix (H-bond between position *i* and *i + 5*) are sometimes present in proteins but the main helical structure remains the α-one [21].

In the α-helical structure, every amino acidic residue determines the rotation of 100${}^{\circ }$ across the helix axis, with a translation of 1.5 along the axis; this determines that a single turn in the helix happens every 3.6 residues, with a pitch of 5.4 for each turn [1].

**Figure 4:**
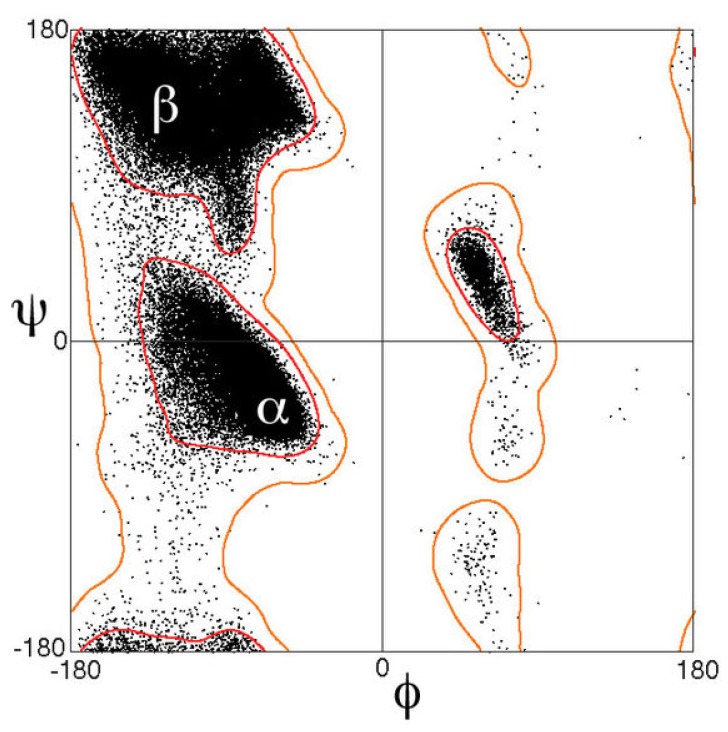
Ramachandran diagram [23].

In order to maintain this conformation, each amino acid has to adopt the dihedral angles of its backbone (φ, ψ) around (−60${}^{\circ }$, −45${}^{\circ }$). Looking at the Ramachandran diagram (Figure 4) it is easy to understand that the sum of the two dihedral angles has to be (more or less) 105${}^{\circ }$ and, as consequence, the angles of amino acid involved in α-helical structures fall along a diagonal stripe, with values that range from (−90${}^{\circ }$, −15${}^{\circ }$) to (−35${}^{\circ }$, −70${}^{\circ }$).

The amino acids are strictly packed in the helix, almost leaving no empty space inside the spiral and they expose their side chain outside, pointing them downward, like a pine tree [22].

Not all peptide chains can assume this conformation, because every amino acid has a different propensity in forming α-helical structures—methionine, alanine, leucine, glutamate and lysine display a very good inclination to form helix, while the presence of proline usually disrupts the helical conformation because of the hindrance of its side chain that forces a bend of about 30${}^{\circ }$ in the helix axis and the lack of the amidic hydrogen necessary to the formation of the H-bond, fundamental for the stability of the structure. However, often proline is found to be the first residue of the α-helix, probably because of the rigidity of its structure [24].

Glycine can also interfere with the formation of α-helix because of its high flexibility. Indeed, the lack of substituents on the α-carbon makes the chain too flexible, enhancing the entropic demand for the formation of an ordered structure as the α-helical one [24].

In fact, the entropic cost is a crucial point for the formation of helical peptides—if this demand is not compensated by enough stabilizing interactions, the folding is not favoured. In order to evaluate if the helical structure is favoured or not it is necessary to also consider the environment in which the peptide is. The helical structure hides the polar amidic bond inside the helix, exposing the (non-polar) side-chains at the external environment—in a polar medium, the internal hydrogen bonds can be easily disrupted by the interaction with the polar solvent molecules, while in a non-polar one, the peptides quickly assume an α-helical conformation, maximising the hydrophobic interactions with the external environment.

The entropic demand can be drastically reduced by the insertion of crosslinks inside the peptide, which stabilize the helical structure and destabilize the unfolded state. So, in order to enhance the stability of α-helical structures, it is possible to modify the side chain of two or more amino acids of the same peptide, by linking them together, forming a stapled peptide that display an helical conformation not only in non-polar environments.

The high hydrophobicity of the helical structure allows the peptide with this conformation to cross the biological membranes. In fact, the α-helical moiety is found to be essential for lots of transmembrane proteins, that are anchored to the membrane surface by a membrane-spanning helix or that possess transmembrane-channels formed by seven helices arranged in a ring structure.

For these reasons, the development of artificial helical peptides is a primary objective in the research of new peptide based drugs, in fact the action of this kind of peptides is not limited to the extracellular environment but can reach a wide variety of new targets by crossing the cellular membranes.

### 2.2. Peptide Properties

#### 2.2.1. Protease Resistance

One of the most interesting properties of stapled peptides is their resistance towards the protease activity [17,25]. The helical structure in fact hides the protease targets, the peptide bonds, inside the spiral structure, preventing the attack of the protease.

The protease resistance often correlates with the degree of α-helical stabilization and with the number of stapling bridges. In fact, proteases require that peptides adopt an extended conformation to hydrolyse amide bonds and so the enhancement of the helicity of the peptide structures can render them protease-resistant. Moreover, a high degree of helical stabilization, even if inducted with other methods such as the use of α,α-disubstituted amino acids, in absence of a staple is not sufficient to display a resistance towards proteolytic degradation—a study by an American research team shows a 9-fold difference in half-life in solution between a stapled peptide and a similar non-stapled one with the same average degree of helicity. This is due to the fact that the presence of a stapling chain not only slows the kinetics of proteolytic digestion but completely removes the possibility of an attack of chymotrypsin in the sites located between the stapling amino acids or immediately adjacent to them [17,25,26].

Stapled peptides also show a good resistance in acidic environments—the insertion of a stapling brace enhances the peptide half-life of even two magnitude orders, allowing the peptide to survive unchanged even to the stomach digestion and so to enter in the blood cycle by intestinal absorption. This property also permits the use of peptides by the oral delivery route, which is one of the preferred routes of administration because of its ease and the high patient compliance for this route.

The half-life enhancement by the insertion of stapling bridges is not limited to the extracellular environment, where the most of proteases are located but it is also registered in the intracellular one [17,25].

#### 2.2.2. Cellular Uptake

As previously mentioned, one of the major limits of peptide drugs is their difficulties in penetrating the cellular membranes. The capacity of cell penetration is directly linked to some peptide properties, such as the hydrophobicity, the helicity and the overhaul charge [17,25,26].

For what is concerning the peptide charge, some data show that negatively-charged peptides rarely cross the cell-membrane, while the penetration is favoured in presence of peptide with a net charge that range from 0 to +2. In order to enhance the cellular permeability of negatively-charged peptides is possible to replace the anionic amino acid of the peptide (Glutamate and Aspartate) with their neutral counterparts (Glutamine and Asparagine) if these are not involved in fundamental interaction with the peptide target. Otherwise, it is also possible to insert positive-charged residues on the N- or C-terminus of the peptide.

Because of the direct dependence of cell-penetration with hydrophobicity and helicity of the peptide, it appears clear that stapling peptides can contribute to enhance the cellular uptake of the peptide. In fact the insertion of a staple enhances the helicity of the peptide by forcing it in an α-helical conformation and also increases its hydrophobicity exposing the non polar side chains to the external environment [17,25,26].

The peptide uptake mechanism is still not completely known. However, some investigations show that probably is involved an energy-dependent pinocytosis, or in few cases a direct cell penetration, especially for Arginine-rich peptides [17].

**Figure 5:**
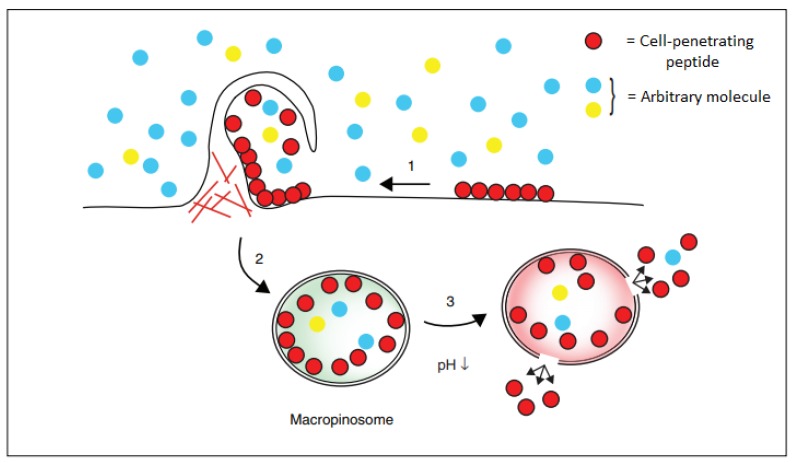
Pinocytosis mechanism [27].

Pinocytosis, also known as fluid endocytosis, consists of the cellular uptake of small molecules by the invagination of the cellular membrane, forming a small vesicle inside the cell, which in the presence of the right conditions can release its load (Figure 5). The pinocytosis mechanism consists of three different steps—in the first, the cell-penetrating peptides bind to the cellular membrane, mainly by polar interaction with the negatively-charged residues of the membrane. Then, the peptides stimulate the cellular uptake by endocytosis that leads to the formation of a vesicle containing the peptides. In the third rate-limiting step, these are released in the cytoplasm by the disruption of the vesicle involving an ATP-dependent process. Even if the precise mechanism of endosomal escape remains unsolved, a pH drop appears to be fundamental for the release of the peptides enclosed in the vesicle [27].

### 2.3. Experimental Determination

In order to assess the potential application of a stapled peptide, it is necessary to evaluate its properties, such as the helicity, the proteolytic resistance, the cellular uptake and the activity.

For this reason, it is fundamental to establish a series of tests in order to evaluate the advantages given by stapling and to make a comparison with other kind of stapling.

#### 2.3.1. Helicity

The easiest method to measure the helicity of a peptide is the circular dichroism—this technique exploits the different adsorption of one of the component (left-handed or right-handed) of circular-polarized light by a chiral compound. As previously mentioned, the α-helix is constituted by a right-handed spiral and so the absorption of the right- and left-polarized light is different. For the assessment of the helicity of the peptide is employed the far-UV circular dichroism (CD) spectrum—in the presence of an α-helix it shows a double minimum about 208 and 222 nm [28].

Another analytical way to determine the presence of a helix is the use of nuclear magnetic resonance (NMR) spectroscopy. The analysis of the chemical shift and the residual dipolar couplings due to the alignment of two atoms can show the presence of a helical structure. Moreover, for more accurate investigations, it is possible to exploit the nuclear Overhauser effect (NOE). This type of experiment allows to determine which atom is spatially located next to another one—because of the interaction between *i* and *i + 4* residues of an α-helix through hydrogen bond, using this spectroscopic method is possible to evaluate the presence of these hydrogen bond interactions so the presence of a helix in the peptide [29].

The most detailed experimental evidence of the presence of an α-helix is the atomic-resolution X-ray crystallography—in fact this technique can provide a measure of the electronic density of a crystal, allowing to see the presence of atom and chemical bonds. Thus permits an easy identification of the structure of the peptide, providing lots of information about the spatial displacement of the atoms.

#### 2.3.2. Solubility

In order to use some of these spectroscopic techniques, such as CD, it is necessary to solubilize the analysed peptide in high concentrations. Therefore, it became necessary to evaluate and, if necessary, optimize the solubility of the peptide. A good evaluation can be afforded by the HPLC elution profile—late-eluting hydrophobic peptides required to be solubilized previously in a non-polar solvent such as DMSO and then can be diluted in water or they may require some structural modifications. It is also possible to dissolve peptides in aqueous buffers at different pH and salt concentrations in order to evaluate how these affects solubility.

Moreover it is fundamental to assess the properties of the peptides in solution—as many other chemical compounds, peptides can aggregate together in clusters that causes a loss of activity. For this reason, even if peptides are usually employed at very low concentrations (nano- or micro-molar), can be useful to evaluate the formation of aggregates at different peptide concentrations. For this purpose is possible to use gel electrophoresis techniques or gel filtration chromatography, which provide a measurement of the molar mass of the species in solution. If an aggregate is detected, it is necessary to lower the peptide concentration, or even to modify its structure as so to prevent the formation of clusters [17].

#### 2.3.3. Proteolytic Resistance

It is possible to test the proteolytic resistance both in vivo and in vitro. The proteolytic stability can be assessed by the confrontation of the stapled peptide half-life with the stability of a non-stapled one. This property can be evaluated by the measurement of the concentration of a peptide after a well-determined amount of time in a protease-containing buffer. It is important that both the stapled and the non-stapled peptide have the same number of chymotrypsin sites—for this reason usually the confrontation is made between the stapled amino acid and the same amino acid, containing the same substituted but not stapled, non-natural amino acids. In vivo tests can be performed both in cells that in more complex organisms, such as mice, by the analysis of the contents of blood stream after oral gavage treatment [17,28,29].

#### 2.3.4. Cellular Uptake

For the evaluation of the cellular uptake of peptides, usually fluorescence techniques are used [30]. A fluorescent tag has to be inserted for this purpose on the peptide by covalent linkage.

There is a great variety of fluorescent probes on the market with different emission wavelenght and chemical properties; the most common employed are usually coumarin, rhodamine and BODIPY derivatives. The choice of which one to use has to be carried out wisely, in order to select a fluorophore with optical properties and solubility compatible with the assay conditions [31]. The fluorescence intensity depends on the intensity of irradiation, the quantum yield of the fluorophore and the medium of analysis. With the help of a calibrating curve it is possible to measure the concentration of the fluorescence tag and, assuming that it is equal to the peptide one, it’s possible to evaluate the concentration of the analysed peptide. This method is used both for live confocal microscopy and for fluorescence scan of electrophoresed lysates. The first technique allows to screen the concentration of peptide in living cells in the time, while the second one permits to check if the peptides have been or not proteolytic digested. For both the analytical methods, it is important to wash the cells after the treatment with peptides, removing the non-absorbed stapled peptides present in the external medium in order to lower as possible the background noise that they generate.

Moreover, it is also important to perform a maximally tolerated dose titration to screen for constructs that disrupt membranes. For this purpose it is necessary to monitor cells by light microscopy, such as by trypan blue exclusion assays, which rapidly indicate if the cell membrane is intact or not by its absence or presence in the cytoplasm, or by LDH release assays, which monitor the appearance of lactate hydrogenases that indicates the disruption of a cell membrane [17,28,29].

#### 2.3.5. Biochemical Activity

In order to check if a stapled peptide possesses the same activity of the relative non-stapled one, some in vitro and in vivo analysis have to be done. Although in vivo tests are fundamental in order to pass to a clinical test of the peptides, it is preferred to start the testing with in vitro assays, which permits a more controlled environment, avoiding as possible misunderstandings about the activity of the compound.

The fastest way to evaluate how the activity of the peptide is modified by te inserption of the stapling is probably the comparison of the binding constant between the stapled peptide and the not-stapled one. Qualitative or semi-qualitative methods such as ELISA, co-immunoprecipitation, or gel shift assays such as SDS-page assays, usually exploit the use a marker and are quite fast and easy to use but they provide only a yes or no answer. Along them there are some quantitative methods that allow to determine precisely the binding constant; the choice of the assay has to be carried out wisely, depending on the nature of the substrate of analysis. For example Surface Plasmon Resonance provides high-sensitivity measurements but the need of immobiliziation of the substrate on a gold surface can limit the application of this technique; moreover changes in the composition of the buffer can cause a variation in the refractive index of the sample, leading to wrong evaluations of the binding constant. On the other side, Isothermal Titration Calorimetry, measuring the heat changes caused by the interactions between the peptide and his substrate can provide a good measurement not only of the affinity but also the thermodynamic values of the bind; however this technique needs large amounts of substrate and analyte and request high concentrations that in some cases can overcome the solubility of the sample. Another possibility is the use of MicroScale Thermophoresis—this technique allows to determine the bind-constant by the evaluation of how the interaction of the analyte and the substrate can lead to change in the quenching of a fluorophore subjected to a thermal gradient. This method requires a small volume of sample and can be performed in any buffer but it needs the insertion of an opportune fluorophore on the substrate.

Besides the bind-constant, the activity of the drug can be assessed with some specific assays, that measure the inhibition or activation of the target substrate of the peptide—for example, for the evaluation of the cell membrane disruption the LDH assay is usually employed [32].

In order to evaluate which stapling appears to be more effective in maintaining the biological activity of the peptide, it is possible to perform a competitive screening of a library of different-stapled peptides, assessing which compounds show the highest activity towards the target molecule.

It is also recommended to optimize the studied peptide by way of computational structure activity relationship analysis, that can help to choose an opportune stapling and to discard methods that will not lead anywhere, saving precious time and chemicals [17,28].

### 2.4. Peptide Stapling

For the stapling of peptide chains, several different reactions are available and permit to link together the side chains of two amino acids of the same peptide. For this purpose, mild reactions are usually involved; the experimental conditions allow the cyclization without interfering with the other functional groups present in the peptide and, at the same time, maintaining unchanged the chiral centre of each amino acid [28].

These reactions should also involve functional groups that can be easily introduced in the amino acid side chain by an easy modification of natural peptide, without disrupting the chirality of the amino acids.

In the last years, several research groups [17,19,20,28,29,33,34,35] tried to apply some different reactions for this purpose, obtaining more or less good results. Here some of the principal stapling techniques employed are listed.

#### 2.4.1. Ring Closing Metathesis

**Scheme 2:**
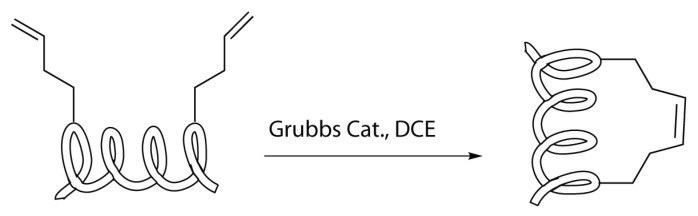
Example of Ring Closing Metathesis Stapling [28].

One of the most promising reaction in peptide stapling seems to be the ring closing metathesis [36]. This reaction consists of the coupling between two terminal alkenes, which leads to the formation of a macrocycle linked by a double bond with the loss of an ethylene molecule (Scheme 2) [17,20,28,29,35].

The reaction is driven by a metal catalyst, that lead to the desired product passing by the formation of a metallacyclobutane intermediate. It is possible to use both ruthenium and molybdenum catalysts:

- First Generation Grubbs Catalysts—this kind of catalyst consists of a ruthenium core substituted with two phosphine groups [usually P(Cy)${}_{3}$], two chlorine atoms and a carbene compound (usually a benzylidene carbene). These metal complexes are quite air-stable and so they are easy to handle [37].
- Second Generation Grubbs Catalysts—these catalysts are very similar to the first generation ones but they bring an *N*-Heterocyclic Carbene (NHC) instead of a phosphine substituent. The insertion of an NHC substituent enhance the activity of the catalyst, maintaining a quite good stability towards air and water [38].
- Schrock Catalysts—these are molybdenum-based catalysts. The electron deficient metal atom is coordinated in a pseudo-tetrahedral sphere by an aryl-imido carbene, a bulky alkylidene carbene and two electron withdrawing alkoxide substituents. These compounds display a great activity but are sensible towards air, water and some compounds with protons on the heteroatoms (-COOH, -SH, ...) [39].

The ring close metathesis usually affords both the E- and Z-isomers. Often, the separation and the geometry assignment of the two isomers may be quite difficult—in order to circumvent this problem it is possible to reduce the double-bond moiety, obtaining a full-saturated bridge. After the RCM reaction, the reduction of the double bond can be carried out with several reagents but it is also possible to perform a one-pot metathesis/reduction tandem process, consisting of the use of a Ruthenium metathesis catalyst along with a reducing agent such as sodium borhydride. In a recent work, Johannes and others showed that the best results in the one-step metathesis/reduction stapling were achieved using triethylsilane as reducing agent, obtaining a yield of 90% [40].

##### Reaction Mechanism

**Scheme 3:**
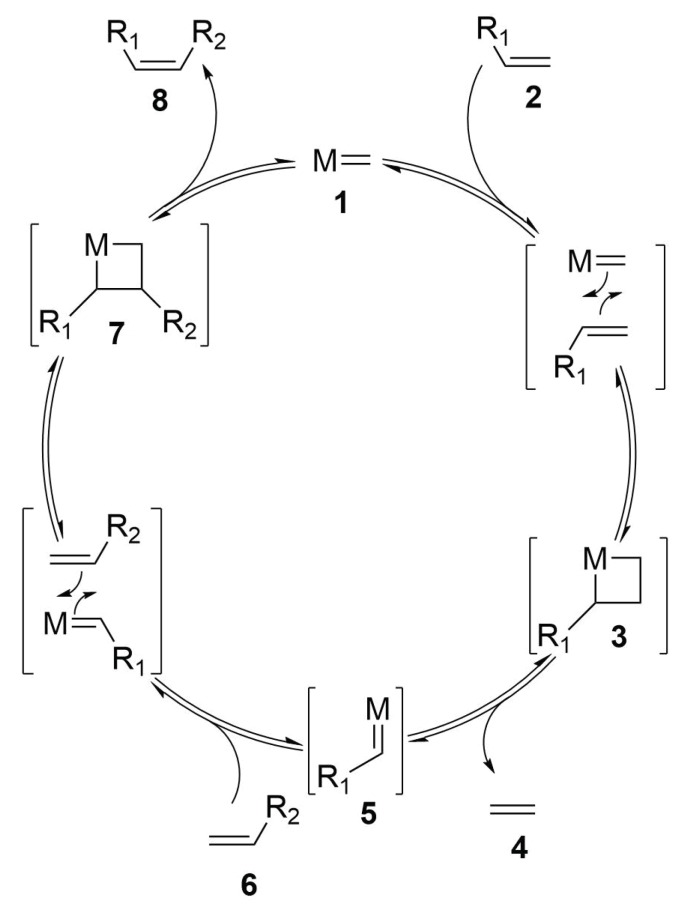
Ring closing metathesis reaction mechanism.

The reaction mechanism is based upon a double [2+2] cycloaddition/cycloelimination between an olefin and the carbene-metal complex (Scheme 3) [41,42]. The first step regards the cycloaddition of the first olefinic compound (**2**) with the metal catalyst (**1**) affording a metallacyclobutane complex (**3**). The thermal [2+2] cycloaddition between olefinic compounds are symmetry forbidden and so require high activation energies. However, the interaction with the d orbitals of the metal atom contributes in lowering the activation energy, allowing the reaction even at room temperature. The second step consists of the cycloelimination of the metallacyclobutane—this [2+2] ring opening can lead to the same starting olefinic compounds (and so the reaction return to the starting point) or can afford a new metal carbene complex (**5**) with the loss of a new olefinic specie, usually ethylene (**4**) or vinyl benzene. Then, the new alkylidene (**5**) incurs in a second cycloaddition reaction with the other olefinic compound (**6**), affording a new metallacyclobutane complex (**7**). The fact that the same molecule possess two olefinic compound that can react together lowers the entropic demand of cycloaddition, affording a single product instead of a mixture of cycloadducts that can lead to the formation of crosslinks between different molecules. The final step consists of the elimination of the coupled olefinic compound (**8**) and the release of the methylidene metal complex that can start a new catalytic cycle.

The distribution of products depend on their energies—olefins possess more or less the same energy and so the cycloaddition can be driven by the reaction condition. In fact, since the main by-product of the reaction is ethylene, it is necessary to remove quickly this gas from the reaction mixture, according with the Le Chatelier effect [41,42].

##### Modified Peptide Synthesis

**Scheme 4:**
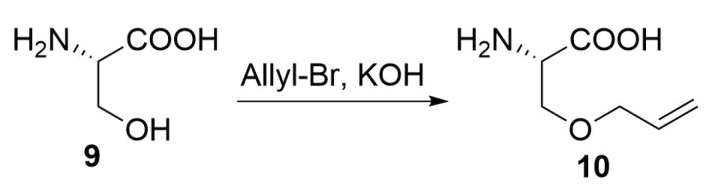
Olefinic amino acid synthesis by nucleophilic substitution.

In order to have the possibility to perform a ring closing metathesis to staple a peptide, it is necessary to have two olefinic-substituted amino acids. Unfortunately, there are no natural amino acids that bring a double bond in the side chain. The easiest way to introduce an olefinic moiety on an amino acid is the allylation of the serine by the nucleophilic substitution reaction between the hydroxyl group of the serine and an allyl halide (Scheme 4) [17].

**Scheme 5:**
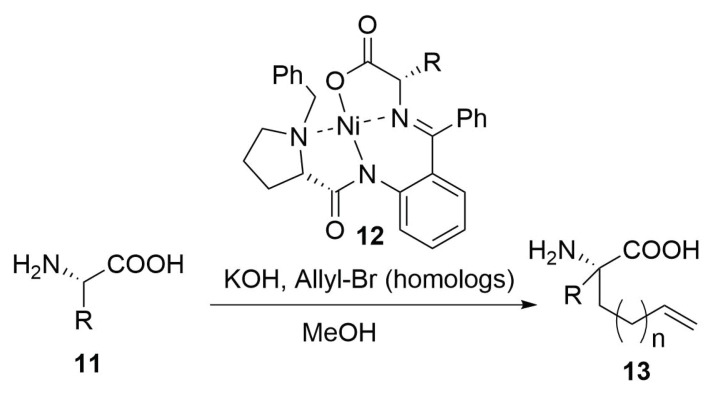
Nickel-catalysed olefinic amino acid synthesis. R = -H, -Me.

Another possible way that has been widely used it is the employment of a chiral nickel catalyst that permit to insert stereoselectively a terminal olefinic hydrocarbon chain on glycine or alanine residues of type **11** (Scheme 5). The substitution reaction is driven by the chiral Schiff base obtained by the condensation between the amine group of the amino acid (Gly or Ala) and the (S)-o-[N-(N-benzylprolyl)amino]benzophenone (BPB). This imine is employed as tetradentate ligand in order to form a Ni${}^{II}$-complex [12,17,28,43].

The metal atom maintain the imine ligand in the right position for a stereoselective omologation, which occur by the extraction of the α-hydrogen of the amino acid by treatment with a base (KOH, ...) and then the stereoselective nucleophilic substitution with an allyl halide or other terminal olefins bringing an halogen on the other side of the chain [43].

#### 2.4.2. Copper Catalyzed Azide Alkyne Cycloaddition

**Scheme 6:**
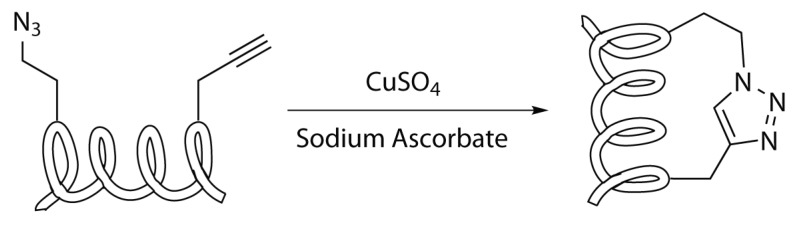
Example of CuAAC Stapling.

The copper catalysed azide-alkyne cycloaddition (CuAAC), usually known as Huisgen Cycloaddition or Click-Chemistry, is one of the important reaction in the field of biological chemistry. This reaction in fact involves functional groups that result to be orthogonal to all the other functionalities present in the cellular environment and the copper catalysis allows mild conditions for the reaction. These properties make the CuAAC reaction also a useful tool for peptide stapling (Scheme 6) [28,29].

The reaction consists of the regioselective [3+2]-cycloaddition between an azide and a terminal alkyne, affording as product a 1,4-disubstituted 1,2,3-triazole ring. The driving force of the reaction is the formation of the triazole ring, which displays a great stability due to the aromatic properties of the ring (ΔG${}^{\circ }$ = −61 kcal mol${}^{-1}$) [44].

##### Reaction Mechanism

**Scheme 7:**
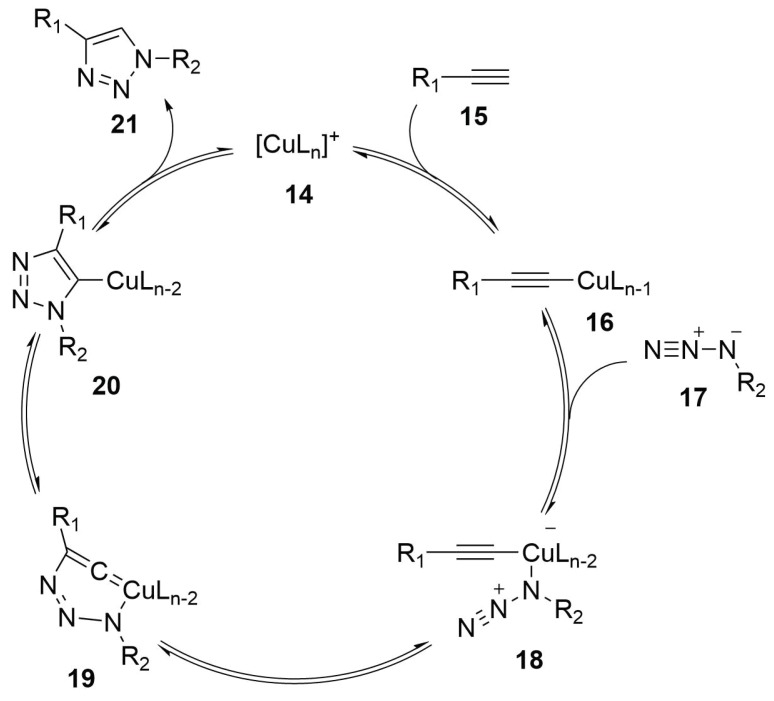
Copper catalysed azide-alkyne cycloaddition (CuAAC) reaction mechanism.

According to the reaction mechanism (Scheme 7), the copper (**14**) links the alkyne (**15**) by a π-interaction and determines the increase of acidity of the terminal hydrogen. The elimination of the terminal proton lead to the formation of a copper-acetylide complex (**16**). Then, the azide group (**17**) is added to the metal atom by a ligand substitution—in this way, the azide group results to be activated and so it reacts with the triple bond forming a triazolic ring (**21**) that is released by the protonation of position 5 [45,46,47].

##### Modified Peptide Synthesis

**Scheme 8:**
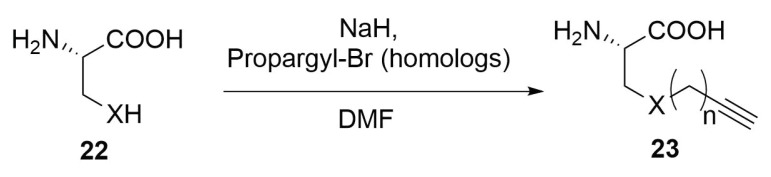
Alkynyl-amino acid synthesis by nucleophilic substitution. X = -O, -S, -COO, -CONH, -CH${}_{2}$COO, -CH${}_{2}$CONH.

For the synthesis of the alkynyl-amino acid, some different synthetic pathways can be followed. The easiest consists of the nucleophilic substitution on a propargyl bromide (or other homologs) by a nucleophilic natural amino acid (Scheme 8). For this purpose it is possible to employ both serine or cysteine residues of type **22**, which can be easily deprotonated by the treatment with a mild base. Glutamate, aspartate, glutamine and asparagine are also suitable to act as nucleophiles in this reaction [48].

**Scheme 9:**
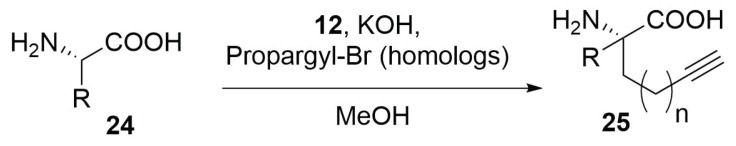
Ni-catalysed alkynyl-amino acid synthesis. R = -H, -Me.

In order to obtain an all-hydrocarbon alkynyl amino acid of type **25**, the same synthetic pathways of the double-bond substituted amino acids can be employed, using the previously showed chiral nickel catalyst **12**, with an adequate alkynyl substrate (Scheme 9) [43,48].

Lots of reaction to insert the azide moiety in amino acids are listed in literature. Here we report a couple of examples to obtain these modified amino acids.

**Scheme 10:**
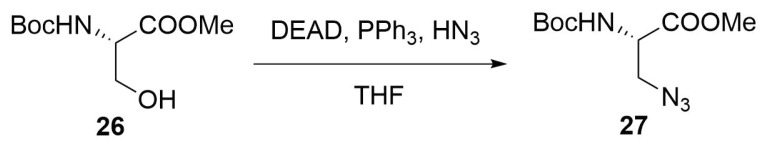
Azide-amino acid synthesis by Mitsunobu reaction.

One possible pathway is the direct insertion of the azide group on a serine residue **26** in the place of the hydroxyl group by the use of Mitsunobu coupling conditions (DEAD, PPH${}_{3}$, HN${}_{3}$, THF) but there is a high risk of racemization (Scheme 10) [48].

**Scheme 11:**
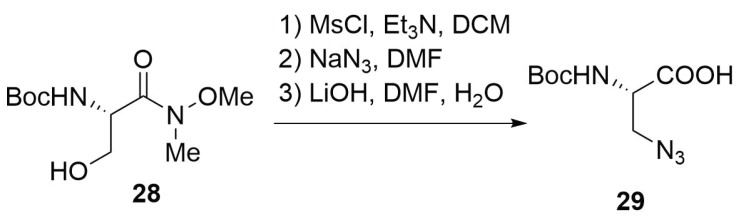
Azide-amino acid synthesis by Weinreb amide.

Another possibility is the mesylation of the serine Weinreb amide **28** and the consequent insertion of the azido group by a nucleophilic substitution on the mesylated hydroxyl group (Scheme 11). This pathway, even if is longer than the other, provides the desired product in high optical purity [48].

**Scheme 12:**
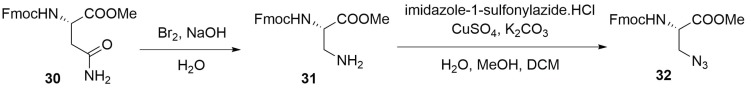
Azide-amino acid synthesis by Hoffmann rearrangement.

Asparagine can also act as a starting material for the synthesis of azido-amino acids **32**—it can incur in a Hoffmann rearrangement, affording the relative amine. Then it is possible to perform a diazotransfer using a reagent such as the imidazole-1-sulfonyl azide that affords the desired product in quite good yields (Scheme 12) [48].

**Scheme 13:**
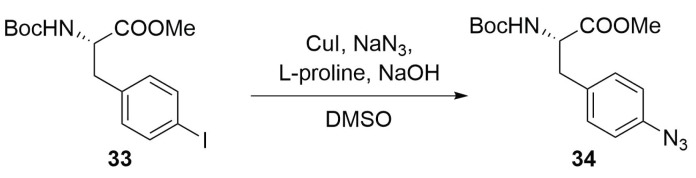
Azide-amino acid synthesis by Ullmann coupling.

The last option we list here is the insertion of the azide moiety in the commercially available p-iodophenylalanine **33** by an Ullmann-type coupling with NaN${}_{3}$ in presence of CuI, that afford the desired azide **34** in good yields (Scheme 13) [48].

#### 2.4.3. Lactamization Reaction

One of the easiest way to form stapled peptides is the formation of an amidic bond between two side chains. In fact, this technique can involve the natural functionalities present on some amino acids such as aspartic and glutamic acid, for what is concerning the carboxylic moiety and lysine for the amine functional group [19,28,29].

**Scheme 14:**
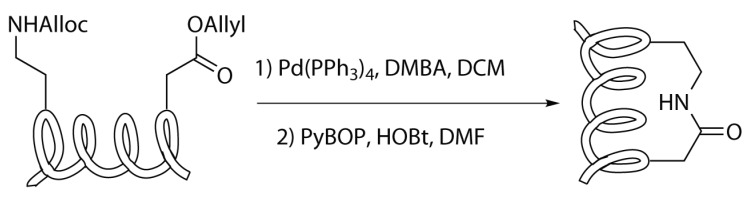
Example of Lactamization Stapling [28].

The lactamization reaction (Scheme 14) is a classical amide formation by the condensation between a carboxylic acid and an amine, with the elimination of a water molecule. However, it is necessary to employ a condensing agent in order to activate the carboxylic group towards the condensation. Several condensing agent are known in literature—they vary from carbodiimides such as DCC and DIC to phosphonium salts as BOP or PyBOP, even to uronium or thiouronium salts as HBTU and TOTT [19].

##### Reaction Mechanism

**Scheme 15:**
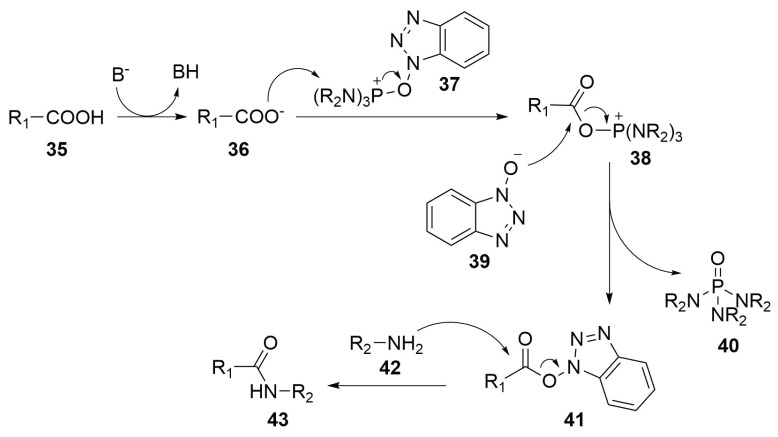
PyBOP/HOBt coupling mechanism.

In order to explain the mechanism of coupling agents, here it is reported, as example, the PyBOP/HOBt (benzotriazol-1-yl-oxytripyrrolidinophosphonium hexafluorophosphate/N-hydroxybenzotriazole) mediated condensation mechanism (Scheme 15) [49].

After the deprotonation of the carboxylic group (**35**) by the treatment with a base, the carboxylate (**36**) attacks the electrophile position of the phosphonium salt (**37**)—the phosphorus atom. This electrophilic substitution lead to the formation of compound **38** with the elimination of a deprotonated N-hydroxybenzotriazole molecule (HOBt) (**39**), which attacks the carbon atom of the carboxylic group of the same compound **38**. The reaction releases a phosphoric triamide molecule (**40**), with the formation of a N-hydroxybenzotriazole ester (**41**). This molecule results to be activated towards the condensation with the amine moiety of the coupling partner (**42**) because of the presence of HOBt as leaving group on the carboxylate. So the reaction proceeds in mild conditions with the nucleophilic attack on the ester moiety that lead to the formation of the desired amidic bond (**43**) with the releasement of HOBt [49].

##### Modified Peptide Synthesis

As mentioned before, this kind of stapling appears to be useful because it is possible to use natural amino acids. However, as in all peptide syntheses, during the synthetic pathway, it is necessary to protect the functional groups involved in the stapling, avoiding undesired coupling with the functionalities of the other amino acids. Hence, the use of protective groups that are orthogonal to the ones used in the protection of the -NH${}_{2}$ and -COOH terminus of the amino acids is compulsory. Considering that the most common protective groups involved in peptide synthesis are the Boc and Fmoc, a good choice could be to protect the side-chain-carboxylate with an allyl group and the amine with an allyloxycarbonyl protective group (Alloc). Both can be easily removed by the treatment with palladium, which maintains unchanged the protective groups on the other functional moieties [19].

#### 2.4.4. Cysteine-Xylene Stapling

Another stapling reaction involving natural amino acids is the m-xylene one, that employ a double nucleophilic substitution on an α,α’-dibromo-m-xylene by the side chain of two cysteine [28,29].

**Scheme 16:**
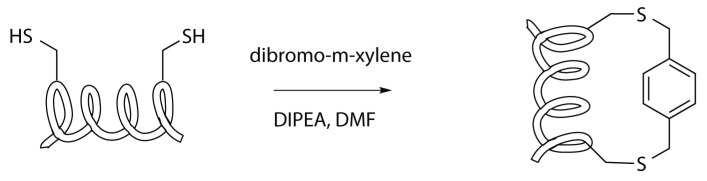
Example of Cysteine-Xylene Stapling [28].

The reaction consists of a simple nucleophilic substitution, where the sulphur atoms of the cysteine residues, after deprotonation with a mild base (DIPEA,... ), act as nucleophiles, attacking the α-carbons of the xylene (Scheme 16).

This stapling represent a stabilization of the disulphide bridges. In fact, cysteines are prone to bind their side chains together, by the formation of a bond between their sulphur atoms. However, these bridges are not very stable and can be easily disrupted, especially in reductive conditions. The insertion of a xylene crosslinker not only contributes to the stabilization of the bridge but also enhance the wideness of the spacer, allowing its use in stapling peptides [28,29].

#### 2.4.5. Cysteine-Perfluorobenzene Stapling

**Scheme 17:**
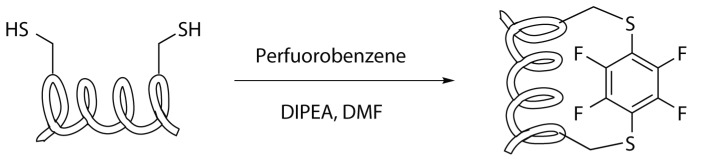
Example of Cysteine-Perfluorobenzene Stapling [28].

Perfluorobenzene stapling is very similar to the m-xylene one—even in this case the stapling involves two cysteine residues but the reaction is an aromatic nucleophilic substitution, with a hexafluorobenzene as substrate (Scheme 17). The fluorine atoms provide a strong inductive electron withdrawing effect that produce highly positive ring-carbon atoms and, in this way, allows the aromatic ring to act as electrophile in nucleophilic substitutions. Moreover, the sulphur atoms have the ability to stabilize the negative charge in the aromatic intermediate species and so, the first substitution with the cysteine functional group leads to increased rate of a second thiolate substitution, favouring the formation of disubstituted species [28,29,50,51].

#### 2.4.6. Thiol-yne/-ene Click Chemistry

**Scheme 18:**
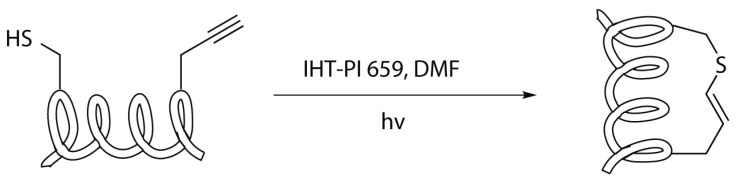
Example of Alkyne/Alkene - Hydrothiolation Stapling [28].

This stapling reaction also involves a cysteine residue. The coupling partner, however, is not another cysteine but a terminal alkyne-substituted amino acid. The reaction consists of a radical addition that lead to the formation of a vinyl sulphide in an anti-Markovnikov way. In order to enhance the rate of reaction it is necessary to employ a radical initiator, that promotes the formation of the sulphide radical. Metal catalyses are also possible but for this application it is preferable to use an UV irradiation, in the presence of a photoinitiator (Scheme 18) [28,34].

It is also possible to use alkene-hydrothiolation reaction to form thioether bridges. However, the absence of the double bond in α-position in respect of the sulphur atom lead to a loss of rigidity in the stapling brace due to an increased structural freedom upon rotation of the C-S bond, with a consequent loss of helicity [29,34].

An advantage of the cysteine stapling is that the chemical properties of staple bridge can be tuned by the oxidation of the thioether to sulfoxide or sulphone. The first one can be obtained by the treatment of the peptide with hydrogen peroxide, while the second requires the addition of performic acid. The oxidation of the sulfur atom lead an enhancement of the polarity of the peptide, specially in the case of the sulfoxide derivative. This stapling technique can be useful to modulate the polarity of high hydrophobic peptides. In fact, as in the case of the lactam stapling, the oxdidation to sulfone or sulfoxide bridge can improve the solubility in water of some low soluble peptides; however, some theoretical calculations show that these staples has a lower desolvation energy than the relative lactam one, consisting of an easier penetration of the cellular membrane. However, the oxidation of the thioether often has a negative effect on the helical content of the structure [52].

##### Modified Peptide Synthesis

**Scheme 19:**
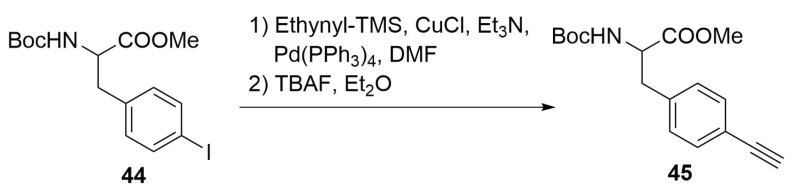
Alkynyl-amino acid synthesis.

As previously reported, for the synthesis of alkyne-substituted amino acids it is possible to follow some different pathways, such as the nucleophilic substitution on propargyl halides or the use of a chiral nickel catalyst [43]. It is also possible to directly insert the triple bond moiety on the aromatic ring of phenylalanine using p-iodophenylalanine **44** as starting material by a Sonogashira cross-coupling (Scheme 19) [53].

#### 2.4.7. Selenocysteine Stapling

**Scheme 20:**
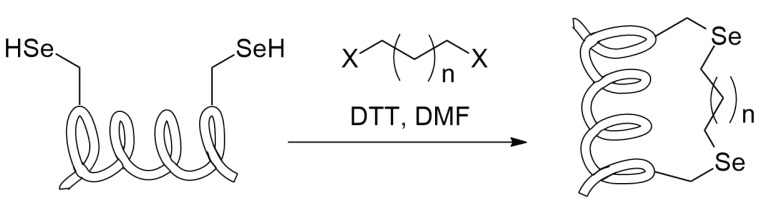
Example of Selenocysteine Stapling [54].

Also, selenoysteine can be employed in the synthesis of stapled peptides. Even in this case a double electrophile can be employed as linker between the two selenium atoms (Scheme 20).

In contrast with cysteine, for which the choice of the linker is quite limited, the higher reactivity of selenocysteine allows the use of a broader range of linker, such as linear dihaloalkanes or dihalopolyethoxy chains and it allows to work in a broader pH range and at milder temperatures. The stapling process is quite simple and it consists of the treatment of the peptide with the electrophilic bridge along with a mild reducing agent as ascorbate or 1,4-dithiothreitol that can be necessary to break the bis-selenide bridges that selenocysteines can form [54].

#### 2.4.8. Tryptophan Condensation

Cysteine and selenocysteine residues are not the only natural amino acids suitable for stapling peptides—tryptophan residues can be exploited for the formation of stapled peptides too [33].

**Scheme 21:**
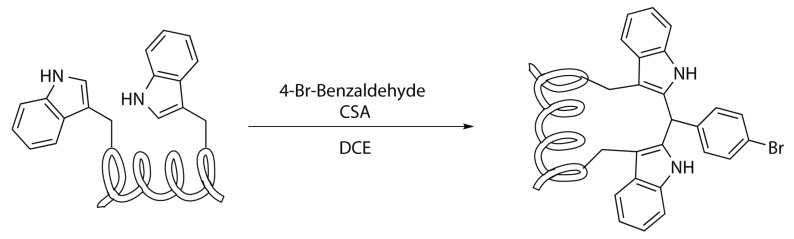
Example of Tryptophan Stapling [33].

These amino acids in fact contain an indole structure, an electron rich aromatic heterocycle. It can be activate towards the coupling with iodophenylalanine or iodotyrosine by the use of palladium-catalysed C-H activation (Scheme 21). However, the use of metal catalysts involves problems about the purification of the product in order to eliminate the toxic metal traces. However it is also possible to employ tryptophan for mild coupling reaction without the use of metal catalysts. This is the case of the acid-catalysed condensation with an aldehyde. Using a double concomitant aldehyde condensation upon the same substrate allows to form a bridge that links the side chains of two tryptophan residues [33].

##### Reaction Mechanism

**Scheme 22:**
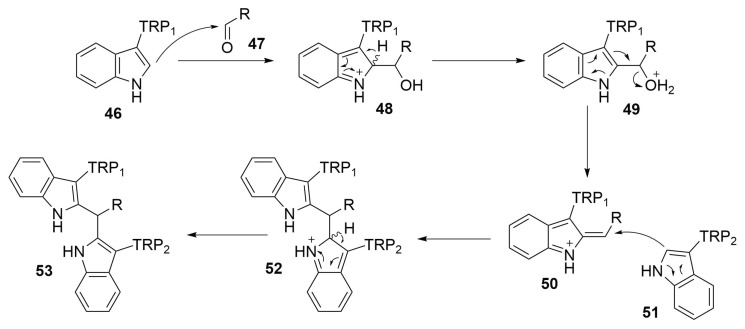
Tryptophan-aldehyde coupling mechanism.

Since the position 3 of the indole ring is involved in the bond with the amino acidic core of tryptophan, the most nucleophilic position results to be the 2 one. Indole is not strong enough to attack aldehydes but in presence of an acid catalysis, it can attack a protonate aldehyde, affording the compound **48**. Then, after restoring the aromaticity by deprotonation, the compound loses a molecule of water, affording the compound **50**. This molecule is attacked by the second tryptophan residue (**51**) on the α-carbon, which after the deprotonation in position 2 restores its aromatic, affording a double coupled tryptophan residues with an aldehyde molecule (**53**) (Scheme 22) [55].

#### 2.4.9. C-H Activation

**Scheme 23:**
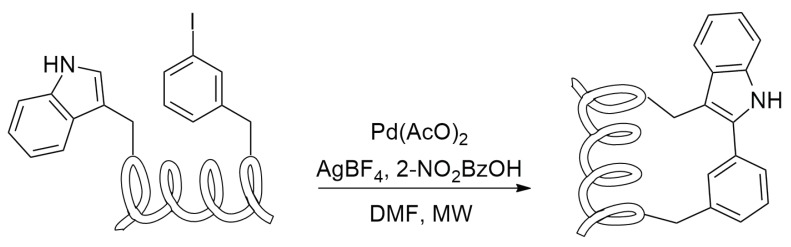
Example of Indole C-H activation Stapling [56].

C-H activation could be useful in the synthesis of tryptophan-containing peptides—a metal catalysis can promove the coupling reaction between the C-2 of the indolic core and the side chain of iodophenilalanine or iodotyrosine (Scheme 23).

Some different conditions and metal catalysts can be employed in the coupling [56,57,58,59,60]; in 2015 Lavilla and others published the first work [56] in which this kind of reactivity has been used in stapling peptides. In their work, they chose to use a palladium acetate catalysis in acidic conditions (AgBF${}_{4}$ 1 eq., *o*-nitrobenzoic acid 1.5 eq.) with microwawe irradiation, obtaining the desidered stapled peptides in very good yields.

This technique can also be employed in the stapling of peptide sequences containing two tryptophan residues by the use of a dihalobenzene as a bridge between the two indole rings.

The main drawback of this kind of satpling is the low conversion yield obtained in presence of peptides containing cysteine, methionine or hystidine residues, probably because their functional groups easy coordinate the palladium core, leading to the metal catalysed hydrolysis of the peptide [56].

In recent years, the direct C(sp${}^{3}$)-H activation has also been employed in peptide functionalization. The use of directing groups is not necessary to reach a good reactivity and regioselectivity, in fact Yu and co-workers [61] demonstrated in 2014 that the peptide itself can act as directing group chelating the palladium atom in a *N,N*-dicoordinated complex, driving the reaction. In 2017, Albericio and his group [62] applied this technique in the synthesis of some staple peptides by the C-H activation of a terminal alanine residue for the cross-coupling with a iodo-phenylalanine derivative with good results.

#### 2.4.10. Future Perspectives on 1,3-Dipolar Cycloaddition for Stapling

At the best of our knowledge there are no publications about the use of the chemistry of 1,3-dipoles in the field of stapled peptides, except for the triazole-stapling.

The comprehensive survey of this area edited by A. Padwa “*1,3-Dipolar Cycloaddition Chemistry*”, appeared in 1984, offers a great panorama on the mechanistic and synthetics aspects of this chemistry [63]. Later on, in 2003, the same editor focused a new volume on the utility of these cycloadditions in the synthesis, including both methodology development and a body of creative and conceptually new applications of these [3+2]-cycloadditions in organic synthesis [64].

**Figure 6:**
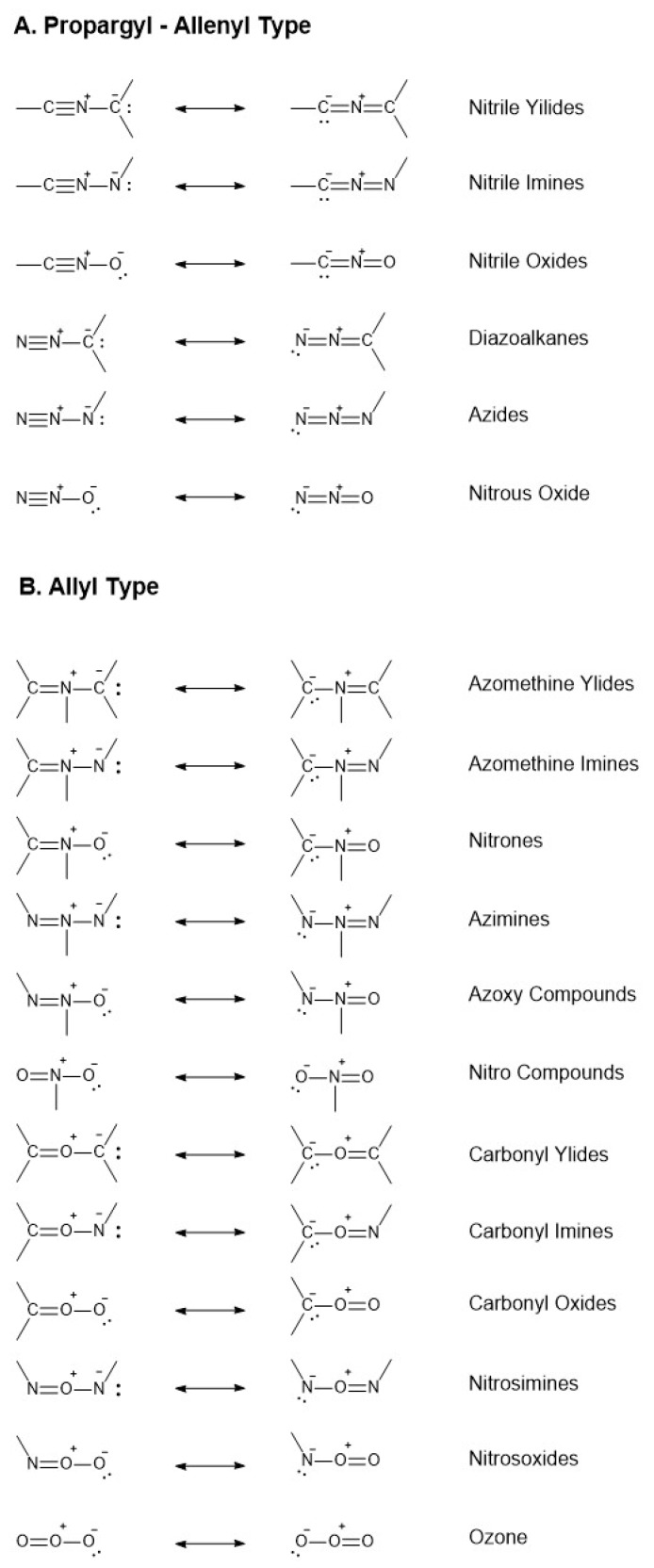
From Huisgen, R. *J. Org. Chem.* **1976**, *41*, 403–419.

1,3-Dipolar cycloaddition reactions, like Diels-Alder (DA) reactions, are [π4s+π2s] reactions and proceed through a 6π-electron “aromatic” transition state (TS) but they differ from the DA reactions in that the 4π-electron component like the allyl anion contain only three atoms, at least one of which is hetero-atom. Cycloadditions to double or triple bonds lead to five-membered heterocyclic compounds. A considerable number of 1,3-dipoles containing various combinations of carbon and hetero-atoms is theoretically possible and many were made. Their reactions whit a variety of dipolarophiles were also investigated both for theoretical and mechanistic investigations and synthetic utility [65]. Restricting the permutations to second row elements Huisgen classified the eighteen possibilities shown in Figure 6, six of the propargyl-allene type and twelve of the allyl type. It is generally agreed that 1,3-dipolar cycloadditions are concerted, like DA. Some 1,3-dipolar cycloadditions are controlled mainly by the HOMO${}_{Dipole}$-LUMO${}_{Dipolarophile}$ interaction and other vice-versa. The smaller the energy gap between the controlling orbitals the faster the reactions. The former are accelerated by electron-donating substituents in the dipole and electron-withdrawing substituents in the dipolarophile. The reversal substitutions are at work in accelerating the second type of cycloadditions. The unique opportunities to play with substituents on both sides of the cycloaddends make these type of pericyclic reactions of valuable importance in the synthesis, offering great advantages in manipulating a variety of structures to reach the desired targets.

As the previous paragraphs showed, the use of 1,3-dipolar cycloadditions is quite restricted to few cases for the preparation of stapled peptides in contrast to large offer of the chemistry of 1,3-dipoles. The main reason for this behaviour of synthetic chemists is certainly due to unfitness of some of the 1,3-dipoles whose structures are reported in Figure 6 to the application in this delicate type of peptide chemistry. Making somewhat a selection, a reasonable criterion is the synthesis of the 1,3-dipole and/or its precursor and the possibility to properly functionalize the dipole itself in view of its application in stapling procedures.

**Scheme 24:**
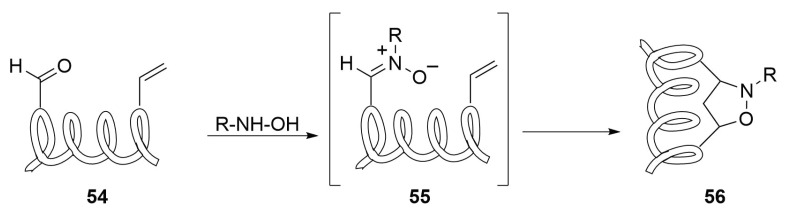
Example of stapling strategy with nitrones.

The allyl type 1,3-dipoles do not offer great possibilities for the case at hand because of their not conventional way of preparation, often requiring complex precursors and specific experimental conditions that must fit with the stability of the peptide chain. Only nitrones can find interesting possibilities in the field and need a solid demonstration. In principle, the strategy is quite simple and straightforward—a suitably functionalized peptide bearing aldehyde and alkene fragments of type **54** (Scheme 24) can be transformed into the nitrone intermediate **55** by reaction with a simple *N*-substituted hydroxylamine allowing for the formation of an isoxazolidine derivative of type **56** through 1,3-dipolar cycloaddition to the olefin moiety.

Other methodologies based on the HgO promoted oxidation of heterocyclic *N*-hydroxylamines to give the corresponding heterocyclic *N*-oxides does not seem practicable because of potential oxidation of other parts of the peptide structure.

Sometimes, nitrones are slow reacting and this could be strong limitation in the stapling step and the reactivity deeply influenced by the nitrogen substituent as well as the stability of the 1,3-dipole itself [66]. According to the FMO theory, nitrones are believed to react with both electron-rich and electron-deficient dipolarophiles; however, if we consider the *N*-methyl-*C*-phenylnitrone, the reactivity sequence is nitroethylene > methyl acrylate > methyl vinyl ether > propene, showing that electron-poor olefins are somewhat the best choice. As we can see, this is an open field that deserves attention and is worth to be explored for the stapling target.

Carbonyl ylides, imines and oxides often require harsh reaction conditions and do not practically fit with the stapling procedures. For example, carbonyl ylides are generated from epoxide ring-opening upon heating even at high temperatures or upon catalysis with transition metals. These conditions are hardly applicable to peptide syntheses [64].

Among the propargyl type 1,3-dipoles, azides have been immediately selected for the ease preparation and functionalization over a variety of structures. Within the same family some 1,3-dipoles at the moment have not been yet considered for their potential application in the field, although in many cases they provide some advantages like the no need of a metal caltalyst in the cycloaddition reaction.

Diazoalkanes or nitrile ylides, for different reasons, cannot find suitable applications. Diazoalkanes can be prepared from alkaline cleavage of *N*-alkyl-*N*-nitroso-ureas, -carboxamides and -sulphonamides as well as dehydrogenation of hydrazones, diazotransfer from azides to active methylenes. Again these methodologies could find difficulties in the peptide synthesis [64]. Similarly, nitrile ylides require thermal photochemical or catalysed methods to be prepared [64].

It is quite surprising that nitrile oxides and imines are still unmentioned in literature for stapling studies. The precursors’ generation relies upon classical and simple nucleophilic addition to carbonyl compounds and a number of modern protocols are reported in literatures for the in situ generation of 1,3-dipoles. Nitriles imines can be prepared from hydrazones with suitable aromatic or aliphatic hydrazines with subsequent chlorination reaction that deserves special attention from the choice of the chlorinating reagent. Classical PCl${}_{5}$ could be detrimental for the core structure of the peptide and other methods such as thermolysis of the sodium salt of α-nitro aldehyde hydrazones or the use of lead acetate to finally cite the photolysis of sydnones do not offer valuable alternatives [67].

On the other side, the simple preparation of nitrile oxides could find interesting application in stapling protocol.

Nitrile oxides can be easily synthesized by the oxidation of oximes **57** or other substrates and incur in 1,3-dipolar cycloaddition with unsaturated compounds, affording heterocyclic rings such isoxazoles or isoxazolidines [68,69,70].

**Scheme 25:**
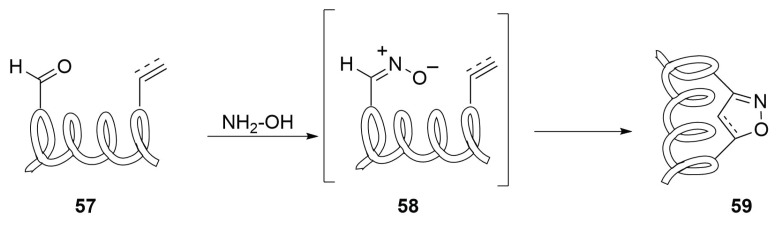
Example of Nitrile Oxide Stapling [28].

The strategy is to use this kind of reactivity in order to bind together a 1,3-dipole-substituted amino acid and an alkyne-one, generating an heterocyclic bridge (Scheme 25). The reaction is usually concerted and it affords a single regioisomer, according with the FMO theory. In fact, for example, in the nitrile oxide-alkyne cycloaddition, the orbitals involved in the reaction are the LUMO of the nitrile oxide and the HOMO of the dipolarophile—the first one has a bigger coefficient on the carbon in α to the nitrogen, while the second one is more expanded on the terminal carbon atom of the alkyne and thus leads to the formation of a single product of cycloaddition.

##### Modified Peptide Synthesis

The possible pathways for the synthesis of the alkynyl-amino acid have been already explored in the previous paragraphs, while for what is concerning the synthesis of the 1,3-dipolar ones, some different synthetic pathways can be considered.

**Scheme 26:**
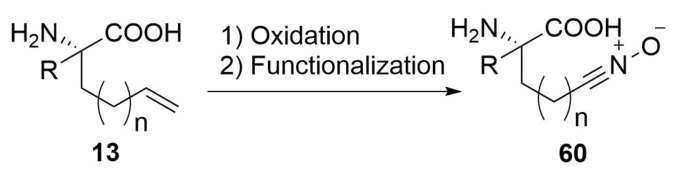
Nitrile oxide-amino acid synthesis from double bond oxidation.

A double bond-substituted amino acid, can be oxidised [71] and then it is easy converted in the relative 1,3-dipole by functional group interconversion (Scheme 26) [72,73].

**Scheme 27:**
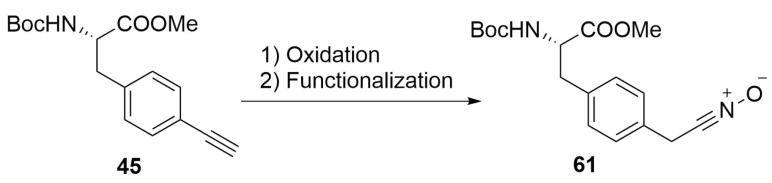
Nitrile oxide-amino acid synthesis by alkyne hydroboration/oxidation.

Furthermore hydroboration of alkyne-substituted amino acid [74] leads to the formation of an aldehydic-amino acid, that can be easily converted in a 1,3-dipole (Scheme 27).

**Scheme 28:**
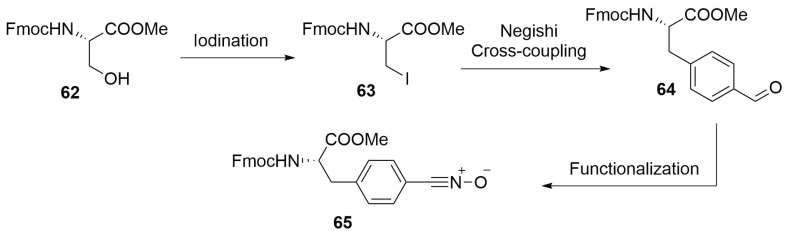
Nitrile oxide-amino acid synthesis by Negishi cross-coupling.

Amino acid bringing an aromatic 1,3-dipole on the side chain can also be prepared starting with the insertion of an iodine atom on a serine scaffold in the place of the hydroxyl group. This halogen atom is necessary to form a zinc-complex, which will be used in a Negishi cross-coupling with a 4-bromobenzaldehyde [75]. The further functionalization of the aldehyde moiety will lead to the desired functional group (Scheme 28).

From simple ideas, it can be seen that the nitrile oxide approach to stapled-peptides is a valuable opportunity to widen and corroborate the pathway towards this type of molecules, opening further scenarios for an easiest preparation of suitably designed biological active compounds.

## 3. Discussion

Making a comparison between the properties conferred to a peptide by a stapling bridge and another is not trivial. Many factors, as the stapling position, the nature of peptide, or the length of the staple brace contribute in determining the properties of the stapled peptide. In the literature, we found only two works in which the authors made a broad comparison between more than two differently stapled peptides—in 2014 Fairlie and co-workers published a deep study on the helicity of some stapled alanine peptides [29], while Li and his group published in 2017 a more extensive evaluation of the chemical and biological effect of stapling on a peptide that targets the estrogen receptor coactivator [28].

**Figure 7:**
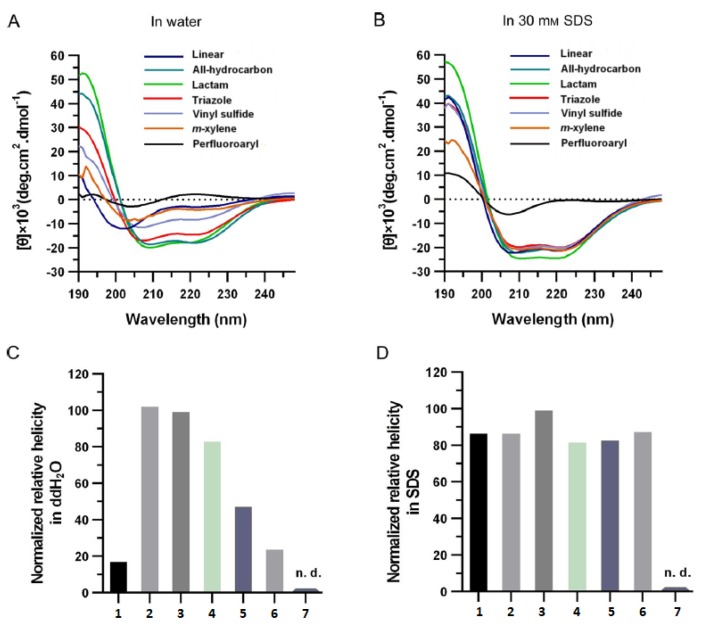
Circular dichroism spectra of peptides measured in ddH${}_{2}$O (**A**) and in 30 mM SDS solution (**B**). Schematic comparison of the relative helical contents in ddH${}_{2}$O (**C**) and 30 mM SDS solution (**D**) (**1** = unstapled; **2** = all-hydrocarbon-; **3** = lactam-; **4** = triazole-; **5** = vinyl sulfide-; **6** = xylene-; **7** = perfluoroaryl-)). The relative helicity was normalized with respect to the peptide with a lactam crosslink. n.d.: not determined as a result of additional absorption [28].

The results obtained of the two different research groups are quite consistent, showing the same general trend for both the classes of peptides (Figure 7). In both cases the circular dichroism analysis performed in a lipophilic medium showed that the peptides have more or less the same helical content of the non-stapled one, which prefers to hide its polar backbone inside the helical conformation, exposing the non-polar side chains. Using water as assay buffer for the CD analysis, the results show that the lactam- and the all hydrocarbon-stapled peptides are the ones that confer the highest degree of helicity. They are followed by the triazole-, the xylene- and finally the sulfide ether-stapled peptide. The vinyl sulfide-stapling confer more rigidity to the structure than the sulfide ether, giving higher values in helicity—these results are confirmed by a previous work [34], in which Li and co-workers determined by NMR spectroscopy and molecular dynamics simulation that, in vinyl sulfide-stapled peptides, the amino acidic residues external to the staple have a certain degree of flexibility, showing more conformational fluctuation.

A deeper analysis was performed with the aid of NMR spectroscopy by Fairlie and co-workers, which evaluated the conformational structure of lactam-, all hydrocarbon- and triazole-stapled peptides in solution.

All these peptides showed low ${}^{3}$J${}_{NHCH_{\alpha }}$ values consistent with helical structures—for the lactam- and the triazole-stapled peptides all the amide coupling constants were <6 Hz except for the values of X${}_{2}$, while the minor grade of helicity of hydrocarbon-stapled peptide was confirmed by a ${}^{3}$J${}_{NHCH_{\alpha }}$> 6 Hz for Ala3 and Ala4.

The peptide containing the lactam crosslink showed stronger αN(*i, i + 3*) and αN(*i, i + 4*) signals in ROESY spectrum, while the all hydrocarbon- and triazole-stapled ones showed more αN(*i, i + 2*) signals—thus indicates a more α-helicity for the lactam-compound, while the other analysed peptides probably have some 3${}_{10}$-helicity in solution.

NMR-derived solution structures for the analysed peptides were calculated and then were compared with the values of an idealized α-helix. The distance between the first and the last α-carbon of the stapled peptides has been assessed to be 5.59 for the lactam-stapled peptide, 5.85 for the all hydrocarbon-one and 5.90 for the triazole-one. The comparison with the usual value for an α-helix (=5.51 Å) showed that the structure of the two latter stapled peptides is slightly more elongated than a common α-helical structure, probably due to a mix with a 3${}_{10}$-helix (=8.30 Å).

The high helical content of the lactam-stapled peptide at first sight could be referred to the presence of an amidic bond on the side chain that allows an additional H-bonding to the backbone. However some assays seem to deny this assumption—first of all, temperature dependence of the chemical shift for the linker amide NH (Δδ/T = 9.3 Hz) was much higher than the characteristic value for a hydrogen bond (Δδ/T ≤ 4 Hz); moreover, the replacement of the amide bond of the staple with an ester one or the methylation of the N-atom of the side chain did not affect the helicity of the peptide [29].

Often, the helicity of peptide is taken as principal measurement to assess which stapling is the best. However Li research group demonstrated that the cellular uptake of the peptide do not correlate with their helical content but with the hydrophobicity, that depends mainly on the polarity of the stapling bridge. In fact the highest hydrophobicity values were recorded for all hydrocarbon- and perfluorobenzene-stapled peptides, followed by the vinyl sulfide- and the xylene-ones, while the most polar triazole- and lactam-stapled peptides displayed low hydrophobicity as in the case of the non-stapled peptide.

**Figure 8:**
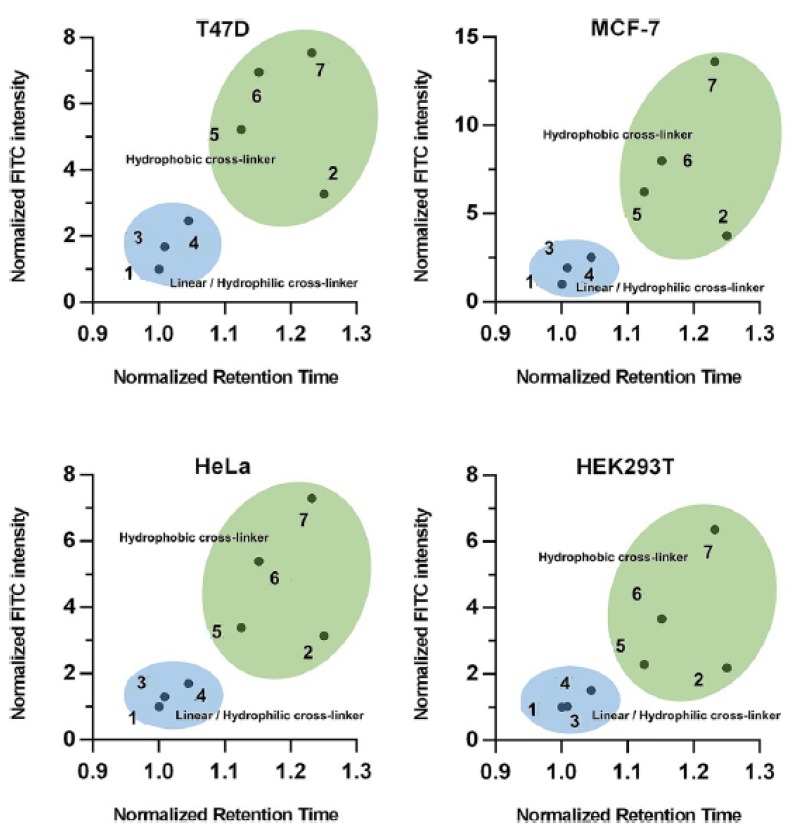
Normalized FAM intensity versus normalized retention time of FAM-labelled peptides **L1b**-**L7b** by using flow cytometry analysis in different cell lines and reversed-phase liquid chromatography. FAM intensity and retention times were normalized with respect to the linear peptide (**1**) (**1** = unstapled; **2** = all-hydrocarbon-; **3** = lactam-; **4** = triazole-; **5** = vinyl sulfide-; **6** = xylene-; **7** = perfluoroaryl-) [28].

The hydrophobicity value represents how well the peptide can mask its polar backbone exposing the non-polar side chains. The expectation was that peptides with a higher helical content show a greater hydrophobicity but, surprisingly, the hydrophobicity values correlate with the polarity of the stapling bridge (Figure 8).

In the case of cysteine-containing staples, as the sulfide ether-one, the hydrophobicity of the external brace can be tuned by the oxidation of the sulfur atom. The transformation into sulfones of sulfoxides enhances the polarity of the bridge, giving an intermediate hydrophobicity value, between the sulfide ether- and the lactam-stapling. Moreover this kind of transformation can have imporant effects on the helicity of peptide—these functional groups confer lower conformational freedom than the sulfide ether, leading to a destabilization of the helical structure [52].

It is interesting that the two sulfoxide isomers display different effect on the helicity of the peptide—in 2016 Li et al. showed that in one of the epimers the S=O bond of the sulfoxide points toward the peptide chain with a dihedral angle of about −81${}^{\circ }$, causing unfavorable steric interactions. This lead to a distortion of the α-helix, with detrimental effects on helicity [52,76].

**Figure 9:**
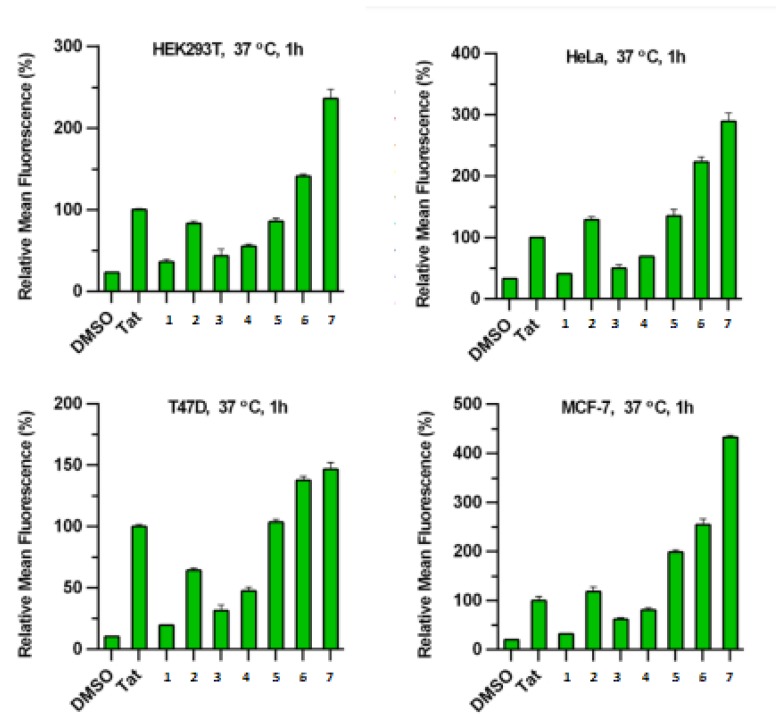
Flow cytometry analysis of stapled α-helical peptides with different cross-links in different cell-lines. Values are normalized with respect to Tat peptide (**1** = unstapled; **2** = all-hydrocarbon-; **3** = lactam-; **4** = triazole-; **5** = vinyl sulfide-; **6** = xylene-; **7** = perfluoroaryl-) [28].

As previously mentioned, both flow cytometry assay and live-cell confocal microscopy have shown that the most hydrophobic peptides (all hydrocarbon- and perfluorobenzene-stapled peptides) have a better membrane permeability, with a higher degree of cellular uptake. Vinyl sulfide- and m-xylene-stapled peptides, although are less hydrophobic, displayed a good cellular uptake, probably due to the greater flexibility of these peptide, that permits conformational changes during the permeation of the cellular membrane. The most polar peptides, as the lactam- and triazole-stapling, although the high helical content, have shown some difficulties in penetrating the membrane and their fluorescence could barely be detected. The data show that the cellular uptake is strictly correlated with the hydrophobic degree of the peptides and so the structure of the stapling-brace play a pivotal role in defining the capability to pass the cellular membrane (Figure 9) [28].

Some research groups [28,77] tried to understand the cellular uptake mechanism for these compounds. The results show that more than one uptake pathway is involved in peptide internalization. The ATP depletion in target cells has a detrimental effect in the cellular uptake, suggesting an energy-dependent pinocytotic mechanism. The treatment with inhibitors of caveolin-mediated endocytosis determines poor effects on the cell permeation, suggesting a caveolin-independent uptake, while the treatment with clathrin-endocytosis inhibitors lead to uncertain results [28,77].

The uptake also seems to involve cell-surface proteoglycans, showing a higher penetration for positively-charged peptides—the membrane penetration increases with the net charge of the peptide chain, but a charge greater than +7 determines a dramatic decrease in cellular uptake [77].

**Figure 10:**
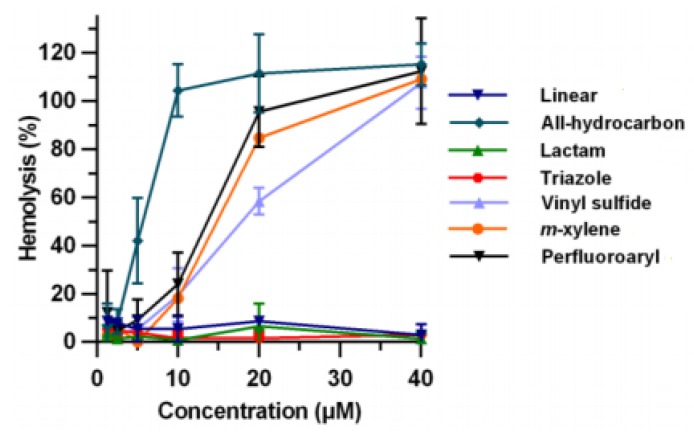
Haemolytic activity of stapled α-helical peptides with different types crosslinks [28].

An increase of hydrophobicity in the peptide structure could lead to side effects for some biological structures. Some assays that have been performed by Li and others showed that the haemolytic activity is strictly correlated with the hydrophobicity of the peptide, consisting of a high degree of toxicity for the all hydrocarbon- and perfluorobenzene-stapled peptides, such as for blood-red cells (Figure 10). HeLa cells also show a high degree of cytotoxicity for the most lipophilic peptides; this is probably due to a destabilizing effect of hydrophobic α-helical peptides on the biological membranes, which leads to the disruption of the membrane itself [28].

Peptides stapled by the insertion of an external bridge between the side chains of two amino acids, as in the case of two azido-amino acid linked by a dialkynyl bridge, have the advantage that their properties can be easily tuned by the choice of the best linking bridge. For example, the net charge of the peptide can be enhanced by the insertion of positively charged bridge, or the solubility can be modulated depending on the polarity of the side brace This allows to tune the peptide properties without the need of make changes in the core structure of the amino acid chain [16]. Moreover it is also possible to insert in the bridge a bioorthogonal functional group for further modifications, such as the insertion of a fluorophore or a recognition element. There is also the possibility to employ asymmetric linkers as a staple—the use of a chiral bridge determines the formation of two diasteroisomers with helicity, cell-permeability and biological activity that can be very different from one isomer to the other [78,79].

For what is concerning the proteolytic stability, there are no evidence that the use of different stapling techniques determines relevant differences in the half-life of the peptide in the cellular environment. In fact, degradative proteolytic enzymes recognize only extended peptide chains and the side brace of the stapled peptides prevent them from entering in the active site of these enzymes [80,81]. However, even if in many cases the use of a staple enhances the resistance towards hydrolysis [17,25,80,81], there is the possibility that the stapled peptides show a life-time shorter than the non-stapled one—for example, in 2017 Chou et al. found that the amino acidic chains outside the staple degrade faster than the unstapled peptide, probably due to a conformational change determined by the staple [82].

## 4. Conclusions

In conclusion, peptide stapling is a useful way to bypass the main problems of peptide drugs by the insertion, in an opportune position of the peptide chain, of a side brace that force the peptide into an α-helical conformation.

The staple enhances the hydrophobicity of the peptide, by hiding the polar backbone of the peptide inside the spiral of the helix and exposing the lipophilic side chains. Thus determines an increase in cellular uptake of the peptide, allowing it to aim not only extracellular targets, but also intracellular ones.

Moreover, the α-helical structure, taken together with the presence of a stapling brace, increase the protease resistance of the stapled peptides, blocking the access of the enzyme to the target sites on the peptide chains.

The enhanced peptide resistance, also showed in an acidic environment and the high lipophilicity of the stapled peptides allow, in some cases, the use of the oral delivery for these peptide drugs, increasing the confidence and ease of use for the patient. In fact, without a staple, most peptides cannot survive stomach digestion and so will never reach or permeate the intestinal mucosal barrier. For these reasons, the most common way to administrate peptide drugs is the parenteral route, but the new findings about stapled peptides seem to have changed this paradigm.

The efficiency of the stapling strictly depends on the position of the staple and the nature of the cross-link. While the choice of the position of the staple mainly depends on the structure of the peptide substrate and cannot be assessed without considering the target of application, the evaluation of the best kind of cross-link could be performed on the basis of previous studies that show the different properties of various typology of stapling.

The results of some comparative studies show that the lactam-staple confers a high degree of helicity to the peptide in aqueous environment, followed by the all hydrocarbon- and the triazole-one. On the other hand, xylene-stapled peptides and sulfide ether-one display a lower degree of helicity.

Usually, the helicity degree is considered the main property of interest in stapled peptides, but the study conducted by Li and al. shows that the cellular uptake of peptides correlates more strictly with the hydrophobicity of the substrate than with its helicity. Moreover, the hydrophobicity of peptides mainly depend on the lipophilicity of the linker and so the nature of the stapling bridge plays a pivotal role in accessing the inner part of cells.

Comparative studies show that the all hydrocarbon-staple is the most hydrophobic, followed by the perfluorobenzene- and the xylene-ones; vinyl sulfide-stapled peptide shows a moderate hydrophobicity, which can be easily tuned by the oxidation into sulfoxides or sulfones, while lactam- and triazole-ones result in being the most polar ones.

However, the same study also shows that the increase of lipophilicity of peptides causes an enhanced cytotoxicity, because of the disruptive interactions with the biological membrane, for both red-blood and normal cells. This determines that, for the bioactive application of stapled peptides it is necessary to establish a good balance between cellular uptake and toxicity, by the tune of the lipophilicity of the external cross-link.

The use of external bridges that bind together the side chains of two amino acids can be useful to have a better modulation of the overhaul polarity of the peptide and its net charge and to further functionalize the stapling brace.

Unfortunately, at the moment a comparative study about the protease resistance given by a different kind of staple has not already been published. At the moment there is no evidence of different behaviours between the different stapling bridges, but further studies should address this.

In conclusion, peptide drugs, with the improvements given by technological developments such as staple-technology, could play a pivotal role in the future in the pharmaceutical field. Stapled peptides allow the targeting of substrate inside cells with a high selectivity and efficiency, with the possibility of accurately tuning the physiochemical properties of these kinds of drugs.

## Figures and Tables

**Table 1:** Non-insulin peptides approved in the years 2000–2016 [6].

Name	Year of Approval	Therapeutic Area	Name	Year of Approval	Therapeutic Area
atosiban	2000	obstetrics	mifamurtide	2009	oncology
taltirelin	2000	CNS	liraglutide	2009	metabolic disease
aviptadil	2000	urology	tesamorelin	2010	antiinfective
carbetocin	2001	obstetrics	lucinactant	2012	pulmnary
nesiritide	2001	cardiovascular	peginesatide	2012	hematology
teriparatide	2002	osteoporosis	pasireotide	2012	endocinology
enfuvirtide	2003	antiinfective	carfilzomib	2012	oncology
abarelix	2003	oncology	linaclotide	2012	gastroenterology
ziconotide	2004	pain	teduglutide	2012	gastroenterology
pramlintide	2005	metabolic disease	lixisenatide	2013	metabolic disease
exenatide	2005	metabolic disease	albiglutide	2014	metabolic disease
icatibant	2008	hematology	oritavancin	2014	antiinfective
romiplostim	2008	hematology	dulagutide	2014	metabolic disease
degarelix	2008	oncology	afamelanotide	2014	dermatology
